# A D-Band Direct-Conversion IQ Receiver with 28 dB CG and 7.3 dB NF in 130 nm SiGe Process

**DOI:** 10.3390/mi14010087

**Published:** 2022-12-29

**Authors:** Fei He, Yuhan Ding, Zhongchen Xu, Menghu Ni, Yibo Tian, Zhenyi Zhang, Zhixiang Shi, Kailei Wang, Qian Xie, Zheng Wang

**Affiliations:** School of Electronic Science and Engineering (National Exemplary School of Microelectronics), University of Electronic Science and Technology of China, Chengdu 611731, China

**Keywords:** D-band receiver, direct conversion, on-chip multiplier chain, SiGe process, sub-THz

## Abstract

In this paper, a D-band direct conversion IQ receiver with on-chip multiplier chain is presented. The D-band LNA with gain-boosting and stagger-tunning technique is implemented to provide high gain and large bandwidth. X9 multiplier chain including Marchand balun and quadrature (90°) hybrid is employed to provide four path LO signal to drive IQ mixer. This receiver is implemented in a 130nm SiGe process and consumes a core area of 1.04 mm^2^. From the experimental results, the proposed receiver exhibits a 20 GHz bandwidth from 150 GHz to 170 GHz, with CG of 28 dB and NF of 7.3 dB at 158 GHz.

## 1. Introduction

Due to the abundant spectrum resources, THz and sub-THz systems attract more and more attention to high-speed communication systems and high-resolution radar [1,2,3,4,5,6,7,8,9]. Especially, with the emergence of advanced silicon processes which offer f_max_ above 400 GHz [10,11], the development of D-band (110–170GHz) front-ends grows rapidly. Nowadays, several D-band transceivers are being reported in communication and radar systems [12,13,14,15].

For the D-band receiver, high gain, low noise, and large bandwidth are the key requirements to strict the building block of the front-ends. To meet these above-mentioned requirements, in this paper, we present a D-band direct conversion IQ receiver with on-chip LO multiplier chain. Benefiting from the on-chip LO chain, this receiver has great potential to be integrated into the digital backend for communication and radar systems.

## 2. Receiver Architecture and Building Block

### 2.1. The Direct Conversion IQ Receiver

**Figure 1:**
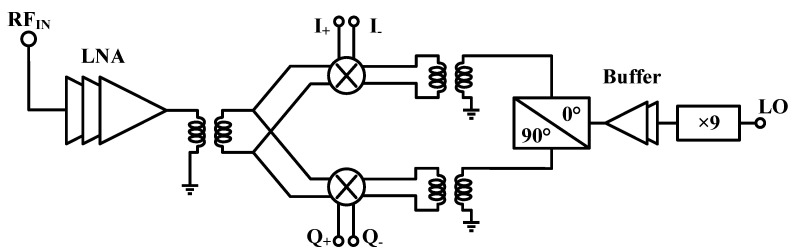
The D-band receiver structure.

The architecture of the proposed D-band direct conversion IQ receiver is shown in Figure 1. It is implemented in a 130 nm SiGe process with an f_t_/f_max_ of 300/450 GHz. This receiver integrated the LNA, LO chain, and IQ mixer. Due to the high gain and large bandwidth of the LNA, the D-band receiver can suppress the noise contribution due to all the blocks after the front-end LNA. To drive the IQ mixer, x9 LO multiplier chain with buffer amplifier, Marchand balun, and quadrature (90°) hybrid are implemented which can provide four paths of D-band signal.

### 2.2. The D-Band LNA

**Figure 2:**
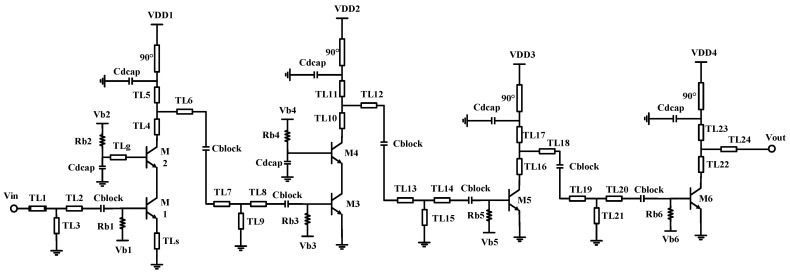
The schematic of the proposed wideband LNA.

The schematic of the proposed LNA is shown in Figure 2. The design of the LNA mainly focuses on optimizing NF, bandwidth, and gain. Firstly, to obtain a sufficient power gain at such high frequency, four stage amplifier is cascaded in which the first and second stages are the cascade structure, with the third and the fourth stages being the common source structure. The sufficient power gain of the LNA is also beneficial to make the contribution of other blocks to the overall chain noise negligible. Secondly, for the NF optimization, since the first stage amplifier dominates the NF of the whole LNA, the source degeneration inductor TL_S_ and gain-boosted inductor TL_g_ are employed in the first stage amplifier, respectively, to obtain a low NF and high gain. Finally, to obtain a wide bandwidth, a gain control stagger-tuning technique is employed in this four-stage amplifier [16,17,18,19]. The peak gain of the first, second, third, and fourth-stage amplifiers is located at 160 GHz, 175 GHz, 145 GHz, and 145 GHz, respectively.

**Figure 3:**
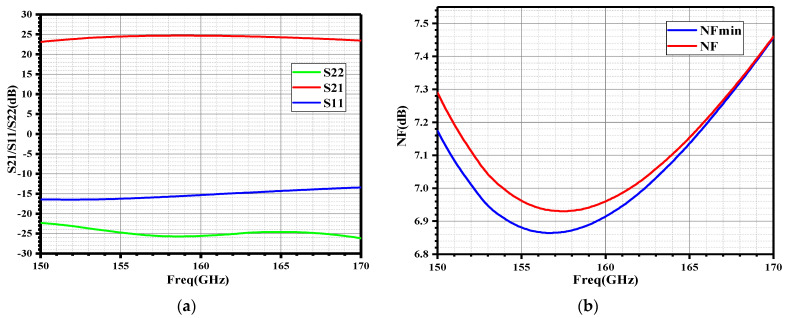
The simulation results of the four-stage LNA: (**a**) S-parameters; (**b**) NF.

The S-parameters and NF simulation results of the proposed wideband LNA are shown in Figure 3. From 150–170 GHz, the proposed LNA exhibits a flat gain range from 23.1 dB to 24.6 dB, it also achieved well input and output conjugate matching. Figure 3b shows the NF simulation result, from 150–170 GHz the proposed LNA exhibits a low NF value range from 6.93 dB to 7.45 dB.

### 2.3. The Mixer

**Figure 4:**
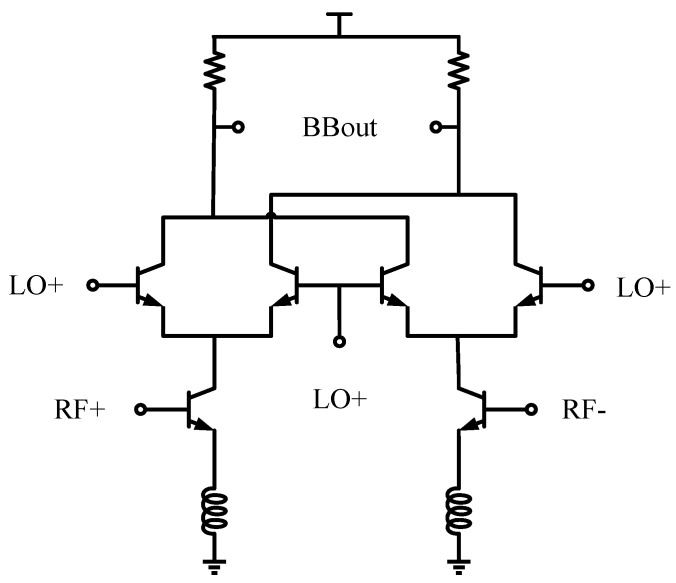
The schematic of the down-conversion mixer.

The schematic of the D-band quadrature mixer is shown in Figure 4; it is based on a pseudo-differential Gilbert cell.

**Figure 5:**
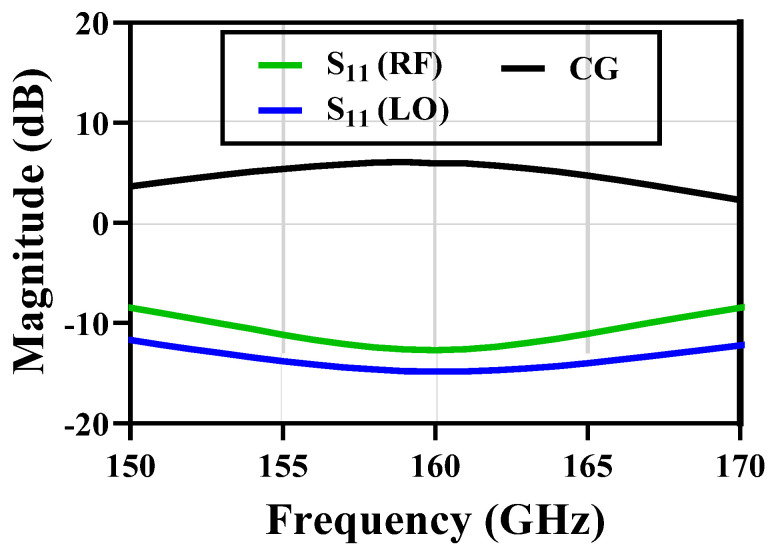
Simulation results of quadrature mixer.

The pseudo-differential transconductance stage eliminates the linearity penalty caused by the tail-current source. As a down conversation mixer, the inductive emitter degeneration technique is employed at high frequency for enhancing linearity. The size of the transconductance stage was determined as minimum as possible to provide a high input impedance to relieve the stress of impedance matching. The size of the switching quad was also determined as a minimum to relax the LO requirement [20]. The mixer drives a 500 Ω load, a relatively large load that provides high voltage gain. Simulating the conversion gain, the S-parameter quadrature mixer results are shown in Figure 5.

### 2.4. The LO Multiplier Chain

**Figure 6:**
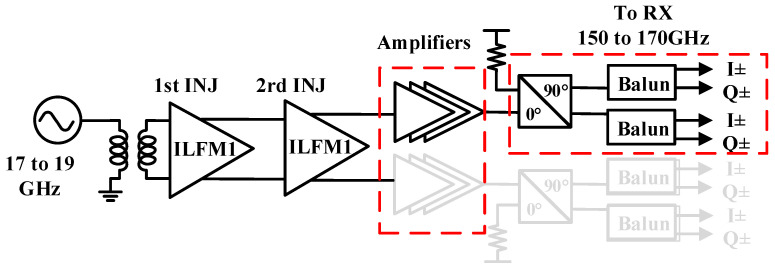
Schematic of the proposed LO chain.

The receiver employs the frequency multiplier chain to generate the 160 GHz LO signal with an external reference of 17 to 19 GHz. The schematic of the multiplier chain with buffer amplifiers is shown in Figure 6.

The core part of the LO chain is a two-stage x3 injection-locked frequency multiplier (ILFM) to produce a x9 signal. Then, three-stage cascade amplifiers, 90° hybrid coupler, and Marchand balun are utilized to generate differential I/Q signals to drive mixers.

**Figure 7:**
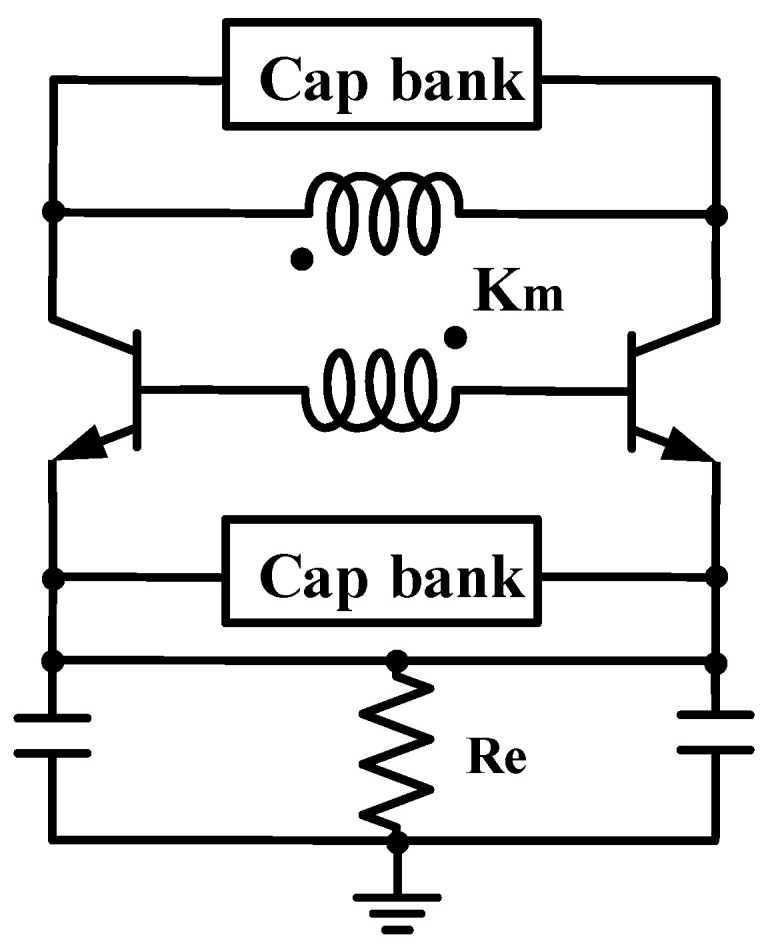
Schematic of the transformer-based VCO with third-harmonic extraction technique.

For the ILFM, Figure 7 represents the schematic of the transformer-based VCO. The x3 ILFM adopts the third-harmonic enhancement technique [21] in transformer-based LC VCO, ensuring the multiplier is working at the fundamental and third harmonic wave. Due to the nature that the frequency is locked at fundamental, with the amplifying and detecting function of the resonant tank in the collector, it is easier for the multiplier to be locked with a wider locking range. As a trade-off between large third harmonic and phase noise, Km is about 0.5 and 0.6 for the transformers of the first and second stages, which use the planar and overlay structure, respectively, to keep a relatively high and constant K_m_ and avoid the inductor self-resonance. The equivalent Q-factor of the transformer in Figure 7 can be calculated as:

(1) $$Q_{eq}=\frac{1+\alpha_{p}\alpha_{s}k_{m}^{2}-\alpha_{p}\alpha_{s}}{\frac{\alpha_{p}}{Q_{p}}+\frac{\alpha_{s}}{Q_{s}}-\alpha_{p}\alpha_{s}\left(\frac{1}{Q_{p}}+\frac{1}{Q_{s}}\right)},$$

where $\alpha_{p}=\omega^{2}L_{p}C_{p}$, $\alpha_{s}=\omega^{2}L_{s}C_{s}$, $Q_{p}$, and $Q_{s}$ are the Q-factor for the primary and secondary winding. When equals to the fundamental and third harmonic, assuming $Q_{p}=Q_{s}$, (1) can be simplified to:

(2) $$Q_{eq}{|}_{\omega =\omega_{fund,3rd}}=Q_{p}\left(1+\frac{2\alpha_{s}k_{m}^{2}}{1+X-2\alpha_{s}}\right),$$

where $X=L_{s}C_{s}/L_{p}C_{p}$. For a transformer based dual tank resonator, when $\omega =\omega_{fund}$, $\alpha_{s}<1$ while $\omega =\omega_{3rd}$, $\alpha >1/\sqrt{1-k_{m}^{2}}$. From (2), it can be concluded that a smaller K_m_ brings a larger Q-factor and impendence at the third harmonic, which is desired for the enhancement and multiplying. However, a large K_m_ is still required for a high Q-factor at the fundamental, because the reduction in K_m_ makes the phase noise performance degrade.

For the core part, the output power at the second amplifier reaches 0.3 dBm at 160 GHz with an input power of 6 dBm. The amplitude and phase mismatch at 150 to 170 GHz is <0.5 dB and <4.5°, respectively, with a –3 dBm input at the third amplifier. The total power consumption is 355 mW, in which the core consumes 91 mW from a 1.6 V supply, while the amplifier chain occupies 264 mW from a 3 V supply.

### 2.5. The Quadrature (90°) Hybrid

**Figure 8:**
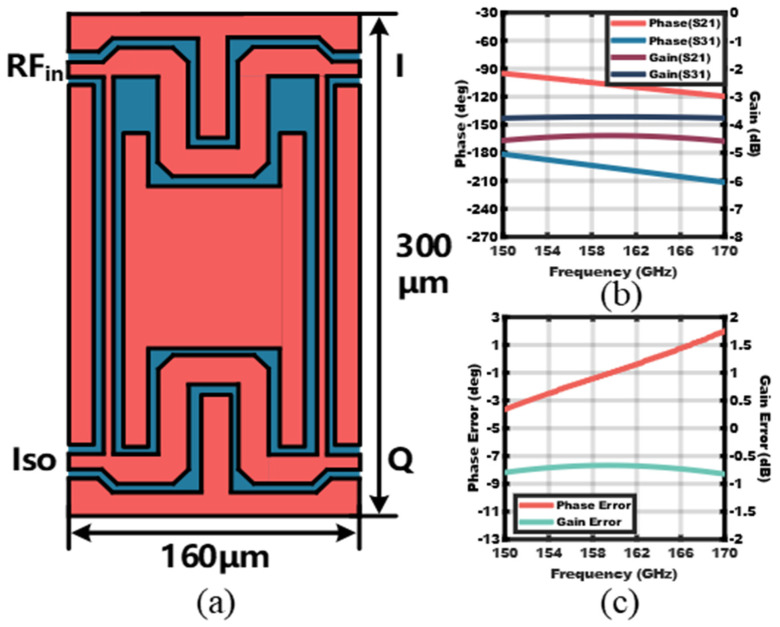
(**a**) Layout of the quadrature−hybrid, (**b**) simulated phase/gain response, and (**c**) simulated phase/gain error.

In the LO chain, to generate the quadrature-hybrid signal, a compact quadrature (90°) hybrid is implemented. The layout of the quadrature-hybrid is shown in Figure 8a. Co-planner waveguides (CPW) are chosen to realize the 50 Ω and 35 Ω transmission lines for their better shielding and higher integration at D-band. The 35 Ω CPWs are meandered to reduce the total size of the quadrature-hybrid. Simulation results show that the insertion loss is 1–1.5 dB and the phase/gain error is within ±3° and ±1 dB over 150–170 GHz.

### 2.6. The Marchand Balun

**Figure 9:**
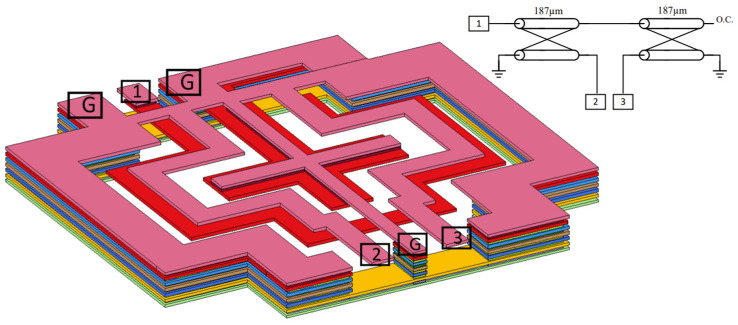
The 3D structure of the proposed Marchand balun.

Marchand balun is employed to achieve the function of single to differential signal conversion. The 3D structure picture and model picture of Marchand balun are shown in Figure 9.

The Marchand balun consists of two couplers. TopMetal2 and TopMetal1 are utilized to generate the coupler and the length of the transmission line in the coupler is 187 µm. The odd and even impedances of the quarter-wave-length coupler in the balun should be 26 and 96 Ω, respectively [22]. To save the chip area, this balun has been folded and it exhibits a symmetrical layout.

**Figure 10:**
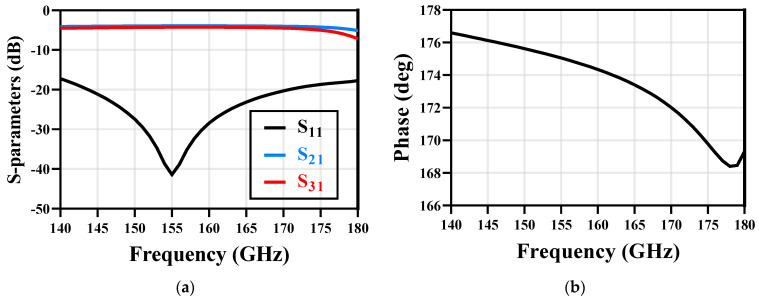
The EM simulation results of the Marchand balun: (**a**) S−parameters; (**b**) phase imbalance.

The proposed balun structure is EM-simulated by a fully-wave EM simulator. Figure 10a shows the simulation results of S-parameters. The S11 of balun is lower than −20 dB and the amplitude imbalance is less than 0.4 dB from 150 GHz to 170 GHz. Figure 10b shows the phase imbalance of the proposed balun; it exhibits a 6° phase imbalance at 160 GHz.

## 3. Experimental Result

**Figure 11:**
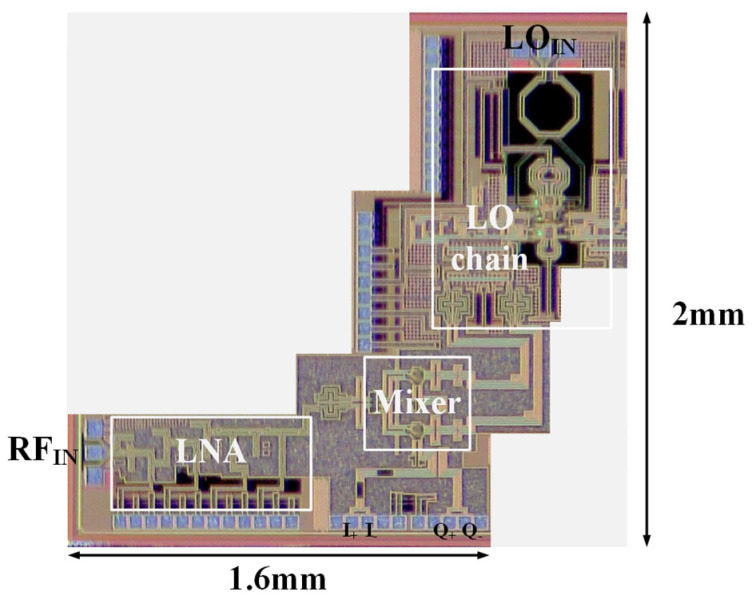
Die photograph of the D−band receiver chip.

The die photograph of the proposed D-band direct conversion IQ receiver is shown in Figure 11; it consumes a DC power of 428 mW and a total area of 1.04 mm^2^ excluding all these RF and DC pads. Due to the measurement limitation, experimental results of conversion gain and linearity of the receiver are presented, with the results of the LNA part being measured and the results of the mixer part simulated.

### 3.1. The Conversion Gain

**Figure 12:**
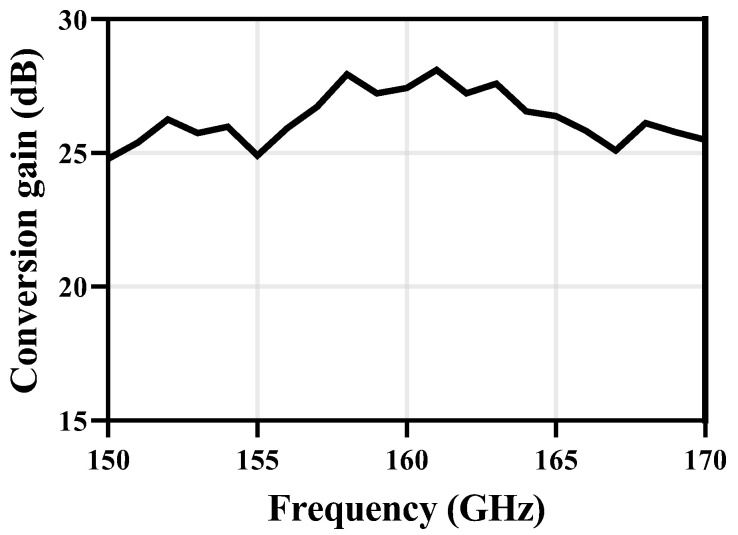
CG results at *f_IF_* = 1 GHz.

Conversion gain results at 1 GHz IF for the proposed receiver are shown in Figure 12. From 150 GHz to 170 GHz, the proposed receiver achieves an overall CG above 25 dB and a maximum CG of 28 dB was achieved at 158 GHz.

### 3.2. NF

**Figure 13:**
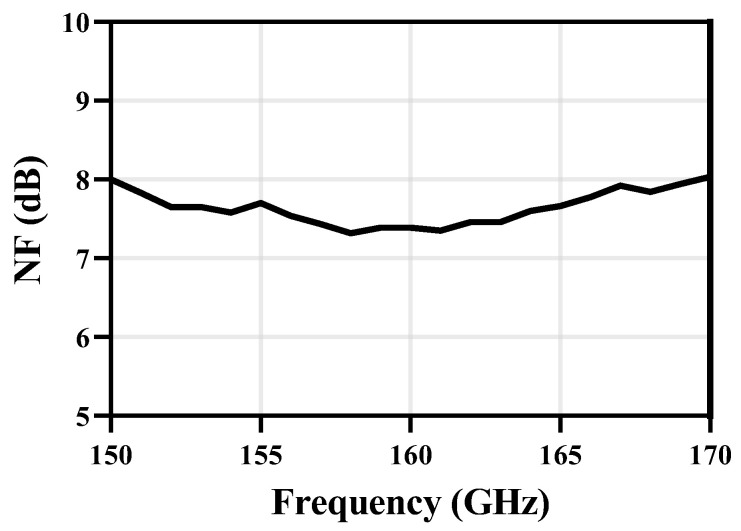
NF results at *f_IF_* = 1GHz.

Due to the low noise and high gain performance of the D-band LNA in the receiver front-end, the proposed receiver exhibits a simulated NF result below 8 dB from 150 GHz to 170 GHz as shown in Figure 13. The lowest NF of 7.3 dB is achieved at 158 GHz.

### 3.3. 1-dB Compression Point

**Figure 14:**
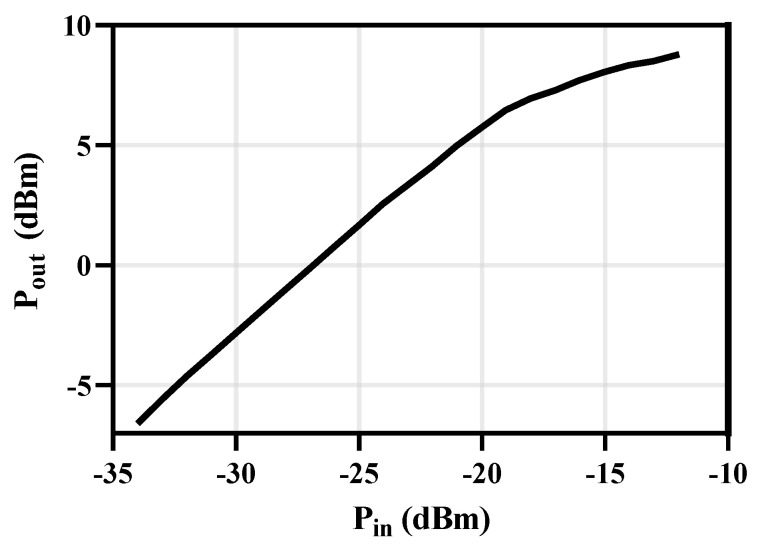
1-dB compression point at 160 GHz.

1-dB compression point results at 160 GHz are shown in Figure 14. The proposed receiver achieved an input compression point of −19 dBm.

### 3.4. S-Parameters

**Figure 15:**
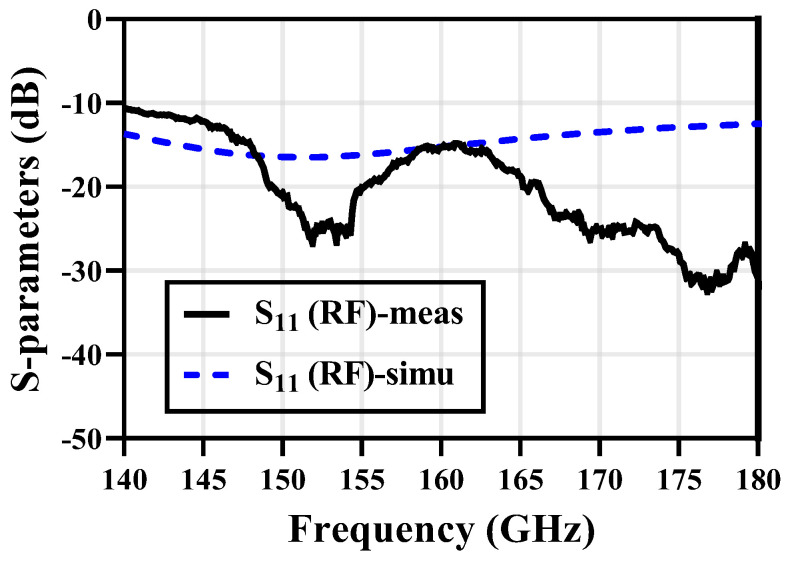
Simulated and measured S-parameters of the RF input.

Measured and simulated S-parameters for the RF input of the proposed receiver are shown in Figure 15. It shows great matching on the RF input. The S_11_ is below –10 dB from 150 GHz to 170 GHz.

## 4. Conclusions

This work presents a D-band direct conversion IQ receiver in a 130 nm SiGe process. The proposed fundamental receiver features a low NF D-band LNA and on-chip LO chain. The performance comparison between our work and the prior silicon-based D-band receiver is presented in Table 1. The proposed receiver is working at the high side of the D-band frequency range with large bandwidth, high gain, and low noise properties. It has great potential for sub-THz communication and high-resolution radar systems.

## Figures and Tables

**Table 1:** Performance comparison and results summary.

	This Work	[12]	[13]	[14]	[15]
**Technology**	**130 nm** **SiGe**	130 nm SiGe	130 nm SiGe	22 nm SOI	22 nm SOI
***f_t_*** **/*f_max_* [GHz]**	**300/450**	250/370	230/280	240/230	240/230
***f*_3_ *_dB_*** **[GHz]**	**150–170**	114–146	112–140	125–145	140–147
**LO generation**	**X9 multiplier chain**	Off-chip	X6 multiplier chain	X9 multiplier chain	X3 multiplier chain
**P_1_** **_dB_ (input) [dBm]**	**−19** ^#^	−7	−24	−30	−29
**CG [dB]**	**28** ^#^	25	27	27	27–30
**NF [dB]**	7.3 *	11.8 *	10 *	8.5 *	9
**P_DC_ [mW]**	**428**	326	280	198	197.5
**Area [mm^2^]**	**1.04**	0.1	1.54	1.44	3.1

* Simulated; ^#^ experimental result.

## Data Availability

The data that support the findings of this study are available within the article.

## References

[1] Chen Z., Ma X., Zhang B., Zhang Y., Niu Z., Kuang N., Li S. (2019). A survey on terahertz communications. China Commun..

[2] Akyildiz I.F., Han C., Hu Z., Nie S., Jornet J.M. (2022). Terahertz Band Communication: An Old Problem Revisited and Research Directions for the Next Decade. IEEE Trans. Commun..

[3] Guan S., Cheng J., Chang S. (2022). Recent Progress of Terahertz Spatial Light Modulators: Materials, Principles and Applications. Micromachines.

[4] Lin C.-H., Cheng Y.-H. (2022). A THz Waveguide Bandpass Filter Design Using an Artificial Neural Network. Micromachines.

[5] Song R., Li N. High Speed Terahertz Communication in the Space and Terrestrial Integrated Next Generation Wireless Communication Systems. Proceedings of the 2020 13th UK-Europe-China Workshop on Millimetre-Waves and Terahertz Technologies (UCMMT).

[6] Stanko S., Caris M., Wahlen A., Sommer R., Wilcke J., Leuther A., Tessmann A. Millimeter resolution with radar at lower terahertz. Proceedings of the 2013 14th International Radar Symposium (IRS).

[7] Kanno A., Sekine N., Uzawa Y., Hosako I., Kawanishi T. 300-GHz versatile transceiver front-end for both communication and imaging. Proceedings of the 2015 40th International Conference on Infrared, Millimeter, and Terahertz waves (IRMMW-THz).

[8] Kang S., Thyagarajan S.V., Niknejad A.M. (2015). A 240 GHz Fully Integrated Wideband QPSK Transmitter in 65 nm CMOS. IEEE J. Solid-State Circuits.

[9] Wang Z., Chiang P.Y., Nazari P., Wang C.C., Chen Z., Heydari P. (2014). A CMOS 210-GHz Fundamental Transceiver with OOK Modulation. IEEE J. Solid-State Circuits.

[10] Heinemann B., Barth R., Bolze D., Drews J., Fischer G.G., Fox A., Fursenko O., Grabolla T., Haak U., Knoll D. SiGe HBT technology with fT/fmax of 300GHz/500GHz and 2.0 ps CML gate delay. Proceedings of the 2010 International Electron Devices Meeting.

[11] Chevalier P., Jungemann C., Lovblom R., Maneux C., Ostinelli O., Pawlak A., Rinaldi N., Rucker H., Wedel G., Zimmer T. (2017). Si/SiGe:C and InP/GaAsSb Heterojunction Bipolar Transistors for THz Applications. Proc. IEEE.

[12] Aguilar E., Issakov V., Weigel R. Highly-Integrated <0.14mm2D -Band Receiver Front-Ends for Radar and Imaging Applications in a 130 nm SiGe BiCMOS Technology. Proceedings of the 2019 IEEE 19th Topical Meeting on Silicon Monolithic Integrated Circuits in RF Systems (SiRF).

[13] Carpenter S., Zirath H., He Z.S., Bao M. (2021). A fully integrated D-band direct-conversion I/Q transmitter and receiver chipset in SiGe BiCMOS technology. J. Commun. Netw..

[14] Farid A.A., Simsek A., Ahmed A.S.H., Rodwell M.J.W. A Broadband Direct Conversion Transmitter/Receiver at D-band Using CMOS 22nm FDSOI. Proceedings of the 2019 IEEE Radio Frequency Integrated Circuits Symposium (RFIC).

[15] Wang C., Rebeiz G. A 2-Channel 136–156 GHz Dual Down-Conversion I/Q Receiver with 30 dB Gain and 9.5 dB NF Using CMOS 22 nm FDSOI. Proceedings of the 2021 IEEE Radio Frequency Integrated Circuits Symposium (RFIC).

[16] Kim J., Buckwalter J.F. (2011). Staggered Gain for 100+ GHz Broadband Amplifiers. IEEE J. Solid-State Circuits.

[17] Jang T.H., Jung K.P., Kang J.S., Byeon C.W., Park C.S. (2020). 120-GHz 8-Stage Broadband Amplifier with Quantitative Stagger Tuning Technique. IEEE Trans. Circuits Syst. I: Regul. Pap..

[18] Karakuzulu A., Eissa M.H., Kissinger D., Malignaggi A. (2021). A Broadband 110–170-GHz Stagger-Tuned Power Amplifier With 13.5-dBm Psat in 130-nm SiGe. IEEE Microw. Wirel. Compon. Lett..

[19] Turkmen E., Burak A., Guner A., Kalyoncu I., Kaynak M., Gurbuz Y. (2018). A SiGe HBT D-Band LNA With Butterworth Response and Noise Reduction Technique. IEEE Microw. Wirel. Compon. Lett..

[20] Turkmen E., Aksoyak I.K., Debski W., Winkler W., Ulusoy A.Ç. (2022). A 225–265 GHz I-Q Receiver in 130-nm SiGe BiCMOS for FMCW Radar Applications. IEEE Microw. Wirel. Compon. Lett..

[21] Liu X., Luong H.C. (2020). A 170-GHz 23.7% Tuning-Range CMOS Injection-Locked LO Generator with Third-Harmonic Enhancement. IEEE Trans. Microw. Theory Tech..

[22] Chen A.C., Pham A.-V., Leoni R.E. (2009). A Novel Broadband Even-Mode Matching Network for Marchand Baluns. IEEE Trans. Microw. Theory Tech..

